# Intestinal ulcers in a patient with myelodysplastic syndrome: a case report

**DOI:** 10.1186/s12876-021-01932-0

**Published:** 2021-10-11

**Authors:** Xiaofen Zhang, Meng Jin, Zhe Shen, Xingyong Wan, Lan Li, Yuwei Zhang, Xinping Zhou, Chen Mei, Hongyan Tong, Chaohui Yu

**Affiliations:** 1 Department of Gastroenterology, The First Affiliated Hospital, Zhejiang University School of Medicine, Hangzhou, 310003 Zhejiang Province China; 2 Department of Hematology, The First Affiliated Hospital, Zhejiang University School of Medicine, Hangzhou, 310003 Zhejiang Province China

**Keywords:** Intestinal ulcers, Endoscopy, Myelodysplastic syndrome, Trisomy 8, Case report

## Abstract

**Background:**

Trisomy 8 positivity myelodysplastic syndrome with Behçet's disease is rare. Isolated trisomy 8 is a frequent cytogenetic abnormality in the MDS, but the characteristic of trisomy 8 and the association between trisomy 8 positivity myelodysplastic syndrome and Behçet's disease is unclear.

**Case presentation:**

Here, we reported a 63‐year‐old man, who presented with fever, abdominal pain and hematochezia. Imaging studies revealed bowel wall thickening and mural hyperenhancement of terminal ileum and cecum. Colonoscopy found multiple round ulcers in terminal ileum, ileocecal valve and multiple yellow dotted pseudomembranous attachments throughout the colon. Capsule endoscopy also revealed multiple irregular ulcers in lower ileum. Serum C-reactive protein levels and fecal calprotectin were abnormally high. The clostridium difficile toxin A and B was positive. However, the patient's intestinal ulcers did not resolve after two weeks course of vancomycin. Considered that the patient was diagnosed as MDS-RAEB2 with a karyotype of 47 XX, + 8. And detailed inquiry of medical history revealed epifolliculitis and frequently recurrent oral ulcers 2 months before admission. A diagnosis of trisomy 8 positivity MDS with BD was made. Then he received glucocorticoid along with the 5th course of azacytidine. The follow-up endoscopy showed significantly improved intestinal ulcer 2 months after treatment. we report a rare disease and provide the diagnose and treatment ideas.

**Conclusions:**

We highlight the challenges and the process of thinking about of the diagnosis. This may provide a new idea for the diagnosis of intestinal ulcers.

## Background

Myelodysplastic syndrome (MDS) is a group of heterogeneous acquired clonal malignant diseases. Isolated trisomy 8 is one of the most frequent cytogenetic abnormalities in MDS which presents in 10–15% of MDS/ myeloproliferative neoplasm (MPN) cases [1]. It has a much shorter median survival compare to normal karyotype. But it is regrettable that the characteristics of trisomy 8 positivity MDS are poorly reported.

Behçet’s disease (BD) is a type of autoimmune vasculitis which characterized by recurrent involvement of the mouth, eyes, genitals and skin. A number of cases have developed gastrointestinal ulcers which called gastrointestinal (GI) BD. Nowadays, some evidence shown an association between MDS, trisomy 8 and Behçet’s-like disease [2].

In our report, we describe a case of trisomy 8 positivity MDS with GI BD and provide the diagnose and treatment ideas. We share the challenges and the process of thinking about of the diagnosis. This may provide a new idea for the diagnosis of intestinal ulcers.

### Case presentation

A 63-year-old man was admitted to our hospital because of fever, abdominal pain and hematochezia for 2 months. He was treated with broad-spectrum antibiotics (ceftizoxime and imipenem) in local hospital. However, symptoms aggravated along with persistent diarrhea. One week later, he was transmitted to our hospital. Nine months before admission, the patient was diagnosed as MDS-RAEB2 and received 4 courses of azacytidine and micro-transplantation. The number of primitive cells in bone marrow declined from 25 to 5% after 4 courses of treatment. There was no family history of autoimmune diseases or inflammatory bowel disease.

On admission, he was conscious and anemic. There was tenderness on palpation of the right lower quadrant with mild rebound pain. Blood tests showed that white blood cells count was 13 × 10^9^/L, hemoglobin level was 82 g/L, platelet count was 93 × 10^9^/L. Erythrocyte sedimentation rate was 53 mm/h (reference, < 18 mm/h), high sensitivity C-reactive protein was 247 mg/L (reference, < 3.1 mg/L). Fecal calprotectin was 872 μg/g.

**Fig. 1:**
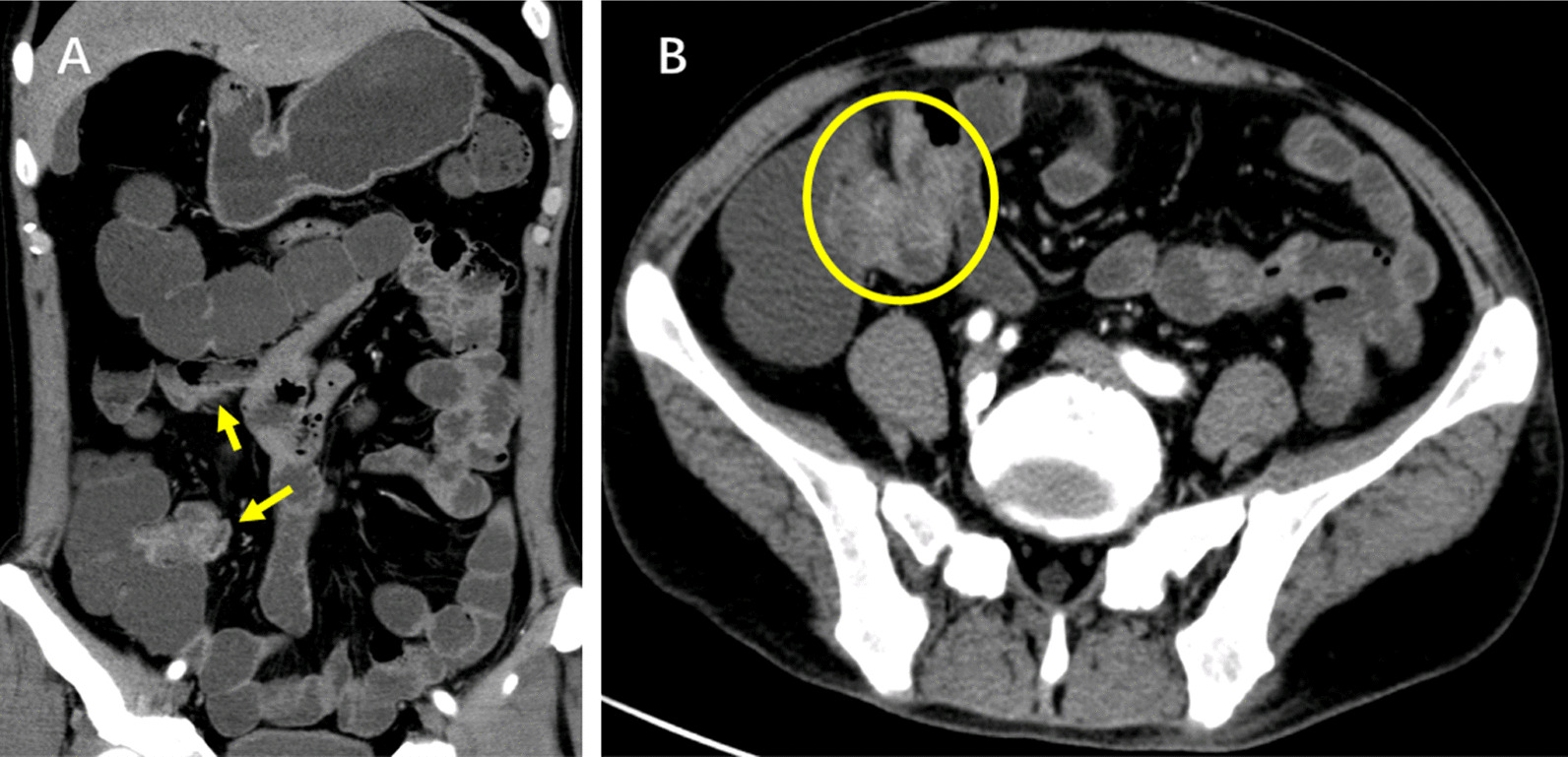
The coronal images (**A**) and cross-sectional images (**B**) of computed tomography enterography showed bowel wall thickening and mural hyperenhancement of terminal ileum and cecum (the arrows)

**Fig. 2:**
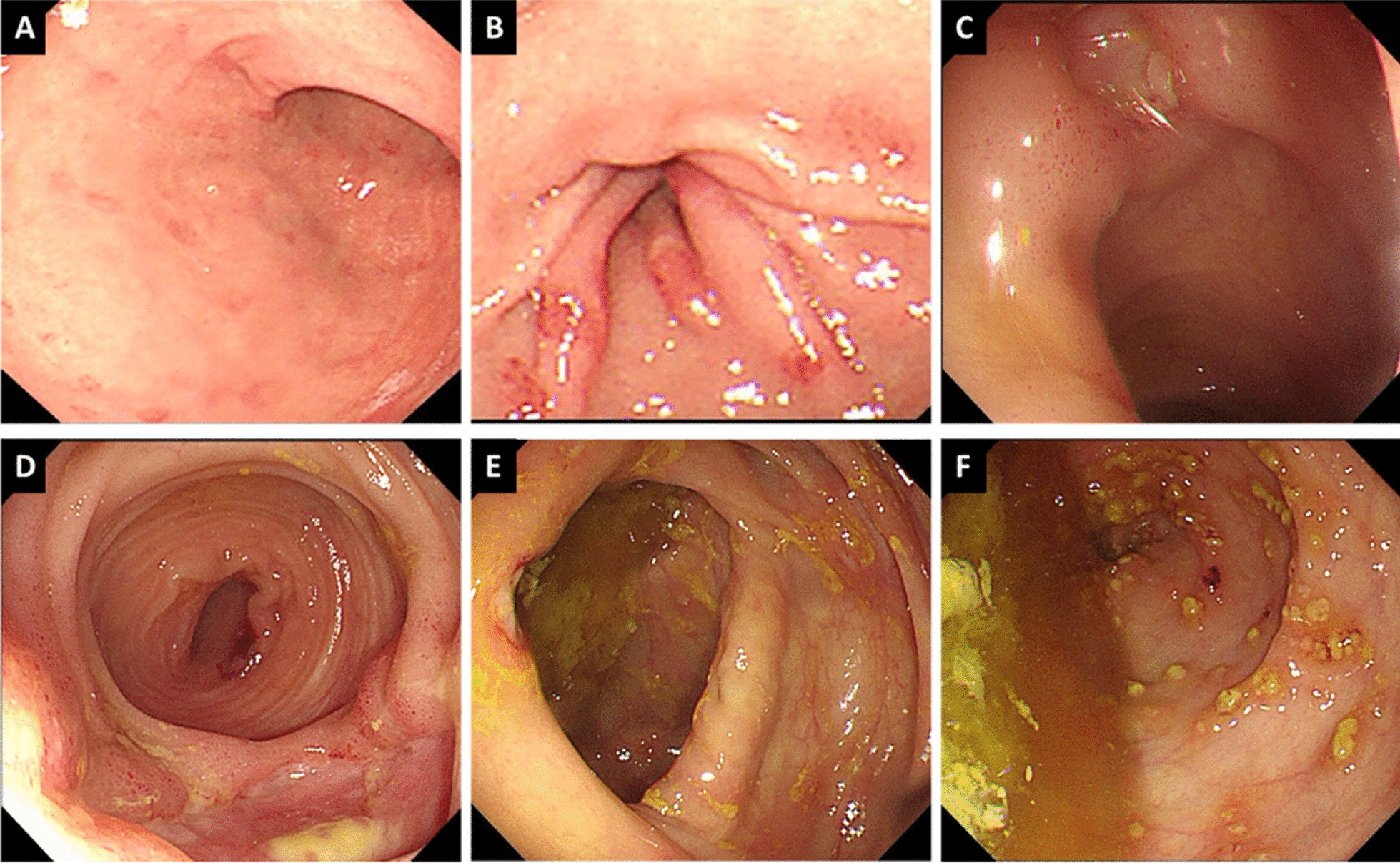
Gastroscopy (**A**, **B**) revealed multiple erosions and telangiectasias in the antrum of stomach. Colonoscopy showed multiple round ulcers in terminal ileum and ileocecal valve and multiple yellow (**C**–**E**) dotted pseudomembranous attachments throughout colon (**F**)

The computed tomography enterography (CTE) (Fig. 1) showed bowel wall thickening and mural hyperenhancement of terminal ileum and cecum. Gastroscopy (Fig. 2A, B) revealed multiple erosions in stomach. Colonoscopy (Fig. 2C–F) showed multiple round ulcers in terminal ileum and ileocecal valve and multiple yellow dotted pseudomembranous attachments throughout colon. Capsule endoscopy also revealed multiple irregular ulcers in lower ileum. Histological analysis showed partially thickened with hyaline degeneration in the wall of small blood vessels.

During multidisciplinary team meeting, diagnosis and differential diagnosis was discussed as infectious colitis, cytotoxic drug-related intestinal ulcer, BD, Crohn's disease and intestinal tuberculosis, etc.

**Fig. 3:**
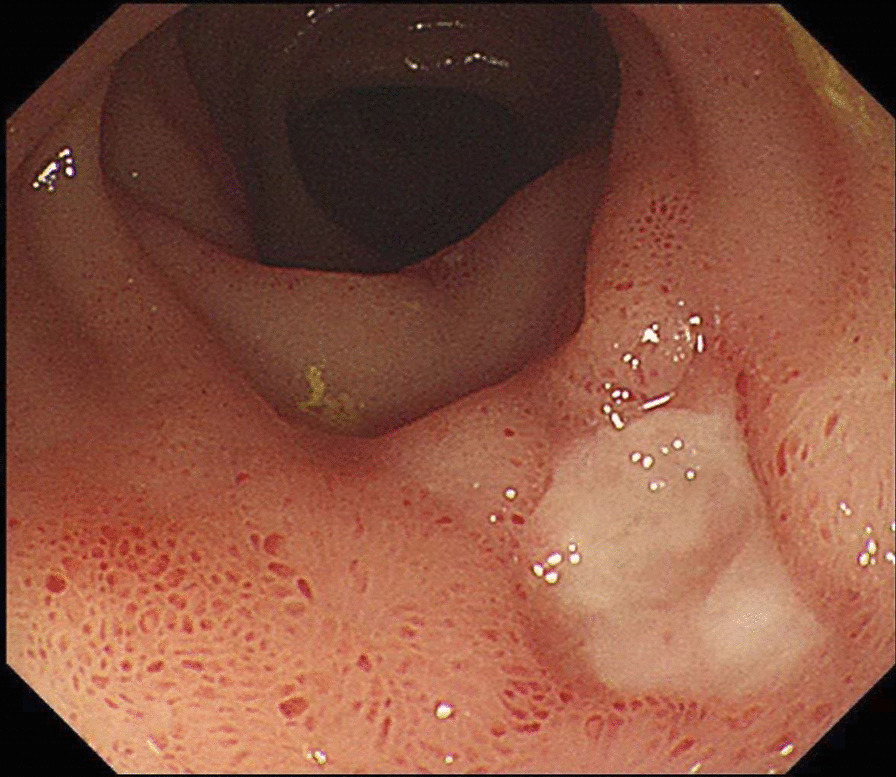
Colonoscopy showed that the intestinal ulcers have no tendency to shrink following successful treatment of clostridium difficile infection

Firstly, stool samples, blood samples and previous colonoscopy biopsy specimen were sent to the laboratory to be tested for infection (including bacteria, tuberculosis, virus and fungus). Intriguingly, the clostridium difficile toxin A and B in stools was positive and clostridium difficile infection was confirmed. The patient received vancomycin 125 mg 4 times per day orally [3]. The fever and diarrhea relieved soon after medication. The test of clostridium difficile toxin A and B were negative after two weeks treatment. Colonoscopy showed that the pseudomembrane disappeared but the intestinal ulcers have no tendency to shrink (Fig. 3). Thus, opportunistic infections not enough to explain the whole state of the symptoms.

Secondly, azacytidine has been reported to induce hematochezia and intestinal ulcer in literature. Since our patient's symptom occurred between the 3rd and 4th course of azacytidine, we assessed the relationship between azacytidine and intestinal ulcer in our case. According to literature, the intestinal ulcers induced by azacytidine occurred shortly after medication and relieved 7 to 20 days after stopping the medication. [4] However, the intestinal ulcers of our patient existed 54 days after last course. The Naranjo adverse drug reaction probability score of this patient is suspicious (2 scores) which not enough to confirm the diagnosis of drug-related bowel disease.

**Fig. 4:**
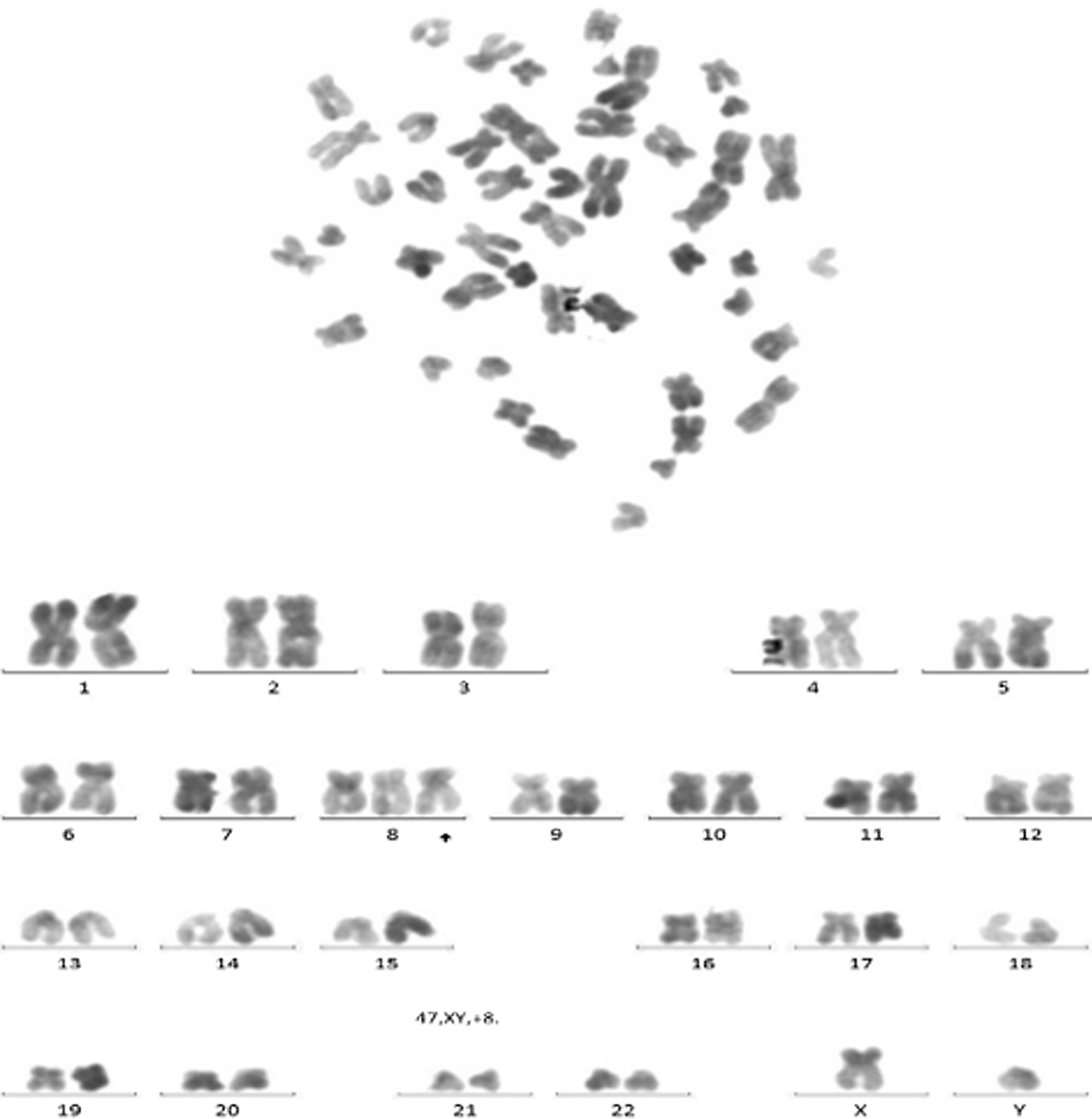
The bone marrow cells analysis of this patient showed a karyotype of 47 XX, + 8

**Fig. 5:**
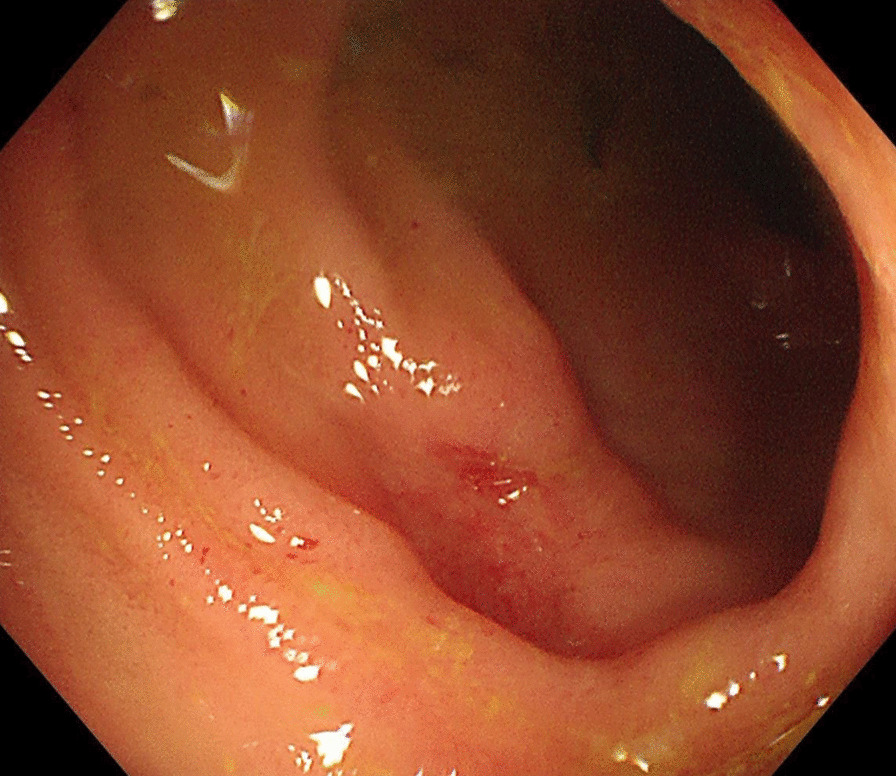
Endoscopy showed significantly improved intestinal ulcer 2 months after treatment

Recently, studies show that trisomy 8 positivity MDS with Behçet’s-like disease often performed as gastrointestinal ulcers. The bone marrow cells analysis of this patient showed a karyotype of 47 XX, + 8 (Fig. 4) accompanied with BCOP, IDH1, PHF6 and RUNX1 gene mutation. Detailed inquiry of medical history revealed epifolliculitis and frequently recurrent oral ulcers 2 months before admission. Based on the International Criteria for BD [5] which point score system that scoring ≥ 4 indicates BD, this patient scored 4. Above all, a diagnosis of trisomy 8 positivity MDS with BD was considered. The patient received glucocorticoid for BD along with the 5th course of azacytidine. Symptoms relieved and follow-up endoscopy (Fig. 5) showed significantly improved intestinal ulcer 2 months after treatment with glucocorticoid.

## Discussion and conclusion

The patient is an elderly man with recurrent abdominal pain and hematochezia. He had been diagnosed as MDS and had received 4 courses of chemotherapy and micro-transplantation. The first appearance of abdominal pain and hematochezia were during the fourth course of therapy. Whether the patient's intestinal ulcer is related to the MDS based on the theory of monism?

Nowadays, evidence showed a high frequency of trisomy 8 and intestinal ulcers are striking features of patients presented with both MDS and BD [2]. But the clinical phenotype, diagnosis, treatments and outcome are poorly reported. So far, only a total of 53 cases about Behçet’s-like syndrome with trisomy 8 positivity MDS were reported in literature [6]. However, none of these reports accurately diagnosed BD from clinical symptoms, imaging and laboratory results. Diagnosis and treatment of these patient remains a challenging clinical task. Thus, prompt and accurate diagnosis of trisomy 8 positivity MDS with BD which involve gastrointestinal ulcers may prevent unnecessary diagnostic procedures.

There are also several limitations in our report. We are unable to explain the association of trisomy 8 positivity MDS and BD. Whether BD as an intestinal performance of trisomy 8 positivity MDS need further verified. The characteristic and the best regimen for treatment of this disease is also unknown. Further studies are needed to determine the underlying mechanisms of trisomy 8.

## Data Availability

Data sharing is not applicable to this article as no datasets were generated or analysed during the current study.

## Abbreviations

MDS: Myelodysplastic syndrome
MDN: Myeloproliferative neoplasm
BD: Behçet’s disease
GI: Gastrointestinal
CTE: Computed tomography enterography
